# Global Research Trends on the Use of Nanotechnology to Boost Meat Production: A Scientometric Analysis

**DOI:** 10.3389/frma.2021.793853

**Published:** 2022-01-13

**Authors:** Emrobowansan Monday Idamokoro, Yiseyon Sunday Hosu

**Affiliations:** ^1^Small-Scale Agribusiness and Rural Non-farm Enterprise, Niche Area, Walter Sisulu University, Mthatha, South Africa; ^2^Department of Economics and Business Sciences, Faculty of Commerce and Administration, Walter Sisulu University, Mthatha, South Africa

**Keywords:** bibliometric study, meat production, global trend, nanomaterials, food security

## Abstract

Meat production plays a vital socioeconomic role for sustainable development and for promoting food security in most countries. However, not much is known about research agendas done globally and the advancement of knowledge-generating networks in this area of study. The present study aims to reveal and analyze scientific research outputs on meat production linked with recent nanotechnology research work done till date. A compilation of research advancement and development within the sphere was realized through a scientometric study to comprehend the trend of research outputs, scientific impacts, authors' involvement, collaboration networks, and the advancement of knowledge gaps for future research endeavors on the current subject matter. Scholarly published articles were retrieved from the web of science (WOS) and Scopus databases from 1985 to 2020 and they were merged together using bibliometric package in R studio. All duplicated articles (438) from both data bases were excluded. A combination of terms (nano^*^ AND (livestock^*^ OR meat^*^ OR beef^*^ OR mutton^*^ OR pork^*^ OR chevon^*^ OR chicken^*^ OR turkey^*^)), and conversely analyzed for scientometric indices. A collection of 656 peer-reviewed, research articles were retrieved for the study period and authored by 2,133 researchers with a collaboration index of 3.31. The research outputs were highest in the year 2020 with total research outputs of 140 articles. The topmost three authors' keywords commonly used by authors were nanoparticles, meat, and chitosan with a respective frequency of 75, 62, and 57. China, Iran, and India ranked top in terms of meat production research outputs linked to nanotechnology and total citation with respective article productivity (total citations) of 160 (3,193), 111 (1,765), and 37 (552). Our findings revealed an increasing trend in research (with an annual growth rate of 25.18%) tending toward advancing meat production with the use of nanotechnology. Likewise, there is an increasing pointer to the fact that research work on nanotechnology and meat production has the prospect to influence positively, decision-making on research direction, and collaborations, hereby increasing the production of meat and its products in the future.

## Introduction

Meat production and especially poultry meat play an essential part in the socioeconomic repositioning for sustainable advancement and in mitigating food insecurity. For instance, it is assumed that by 2020, the poultry sector is expected to provide 40% of the world's sum total animal protein, where the main demand being in the developing world. With meat consumers requesting higher quality meat and its products at cheap prices and increasing competition, the meat production industry has experienced a brilliant change in not only the ingredients, but also the processing technology of meat (Weiss et al., 2010). According to Young et al. (2013), the increase in demand for viable production of meat and its products and the importance on human health and wellness have additionally steered the advancement of innovation in the meat industry. Thus, hopes have increased regarding the utilization of constituents and additives with enhanced functionality to improve the image and quality of meat (Olmedilla-Alonsoa et al., 2013). Several of the widely utilized condiments for improving meat quality and production include, among others, curing agents (sodium erythorbate, sodium nitrite, and nitrate), thickeners (e.g., gelatin), antioxidants [e.g., butylated hydroxytoluene (BHT), butylated hydroxyanisole (BHA), and tocopherols], flavor enhancers (e.g., monosodium glutamate), binders (e.g., carrageenan, sodium caseinate), sweeteners (e.g., corn syrup), tenderizing enzymes (bromelin, ficin, and papain), and humectants (e.g., sodium salt, glycerine) (USDA, 2008; Falowo et al., 2014).

Even though some of the aforementioned food additives are still currently in extensive use in the meat industry, rising health fear has triggered a change in the attention toward the advancement of innovative meat products with lesser quantities of harmful substances [such as sodium salts, saturated fats, cholesterols, and color fixatives (e.g., nitrites)], together with the high use of ingredients shown to possess positive useful benefits on human health. It is, however, anticipated that novel meat and its products processed with new ingredients and processing technologies should possess similar consumer acceptability such as taste, aromatic effects, and visual appeasement as the customary meat products that consumers eat.

In recent years, the meat industry is making great efforts to improve hygiene, extend the shelf life of products, prevent food-borne illnesses and contamination by chemical and physical agents, and to improve their detection and control if contamination has already occurred. As a result of this, there is a constant search for new technologies that can help in achieving these goals. Nanotechnology is one of the major innovations that have already been applied in many different areas/fields with records of several successes to advance science with better product outputs. Results of previous studies show that the use of nanotechnology provides a number of opportunities to improve processes of production, packaging, distribution, and storage of meat (Ramachandraiah et al., 2015; Singh et al., 2016). The applications of nanotechnology presently used for meat production usually comprise the use of nanomaterials/nanoparticles as additives/ingredients that are added directly into meat and its products, or they could be a part of meat packaging/processing techniques (Lee, 2010; Chaudhry and Castle, 2011; Duncan, 2011; Cushen et al., 2012; Azeredo, 2013; Rhim et al., 2013).

Although, the evaluation of scientific outputs has been done for meat production as a sole topic and the same has been carried out for nanotechnology as a sole topic (Laherto, 2010; Wickson et al., 2010; Falowo et al., 2014; Aleixandre-Tudó et al., 2020). However, to the best of our knowledge, no published studies have analyzed scientific literature on meat production in the context of applying the techniques of nanotechnology. Therefore, the present study was carried out to assess and analyze research outputs on meat production *via* the use of nanotechnology. The procedure often adopted to assess and analyze scientific work done on a particular subject matter is referred to as scientometrics or bibliometrics, which is somewhat dissimilar from systematic assessments and reviews of literatures. The foremost objective of scientometric analysis is to analyze trends of research, main research themes, top-cited articles in the field, global, national, and local impact, scientific contributions, and vital actors in a particular field. The bibliometric tool has been utilized to evaluate scientific researches in different fields of endeavors such as medicine, microbiology, geography, among others (Ekundayo and Okoh, 2018; Zyoud et al., 2019; Orimoloye et al., 2020).

## Methodology

### Data Retrieval

The present study made use of published scientific articles on meat production link with nanotechnology research outputs, which were gotten from the combination of Web of Science (WOS) and Scopus archive on June 11, 2021. These databases hosted trustworthy and efficient high-impact scientific studies (Mansoori, 2018; Repiso et al., 2018; Orimoloye et al., 2020). Therefore, in the current study, WOS and Scopus were utilized to realize the required objective. The advanced search function in WOS and Scopus was used due to the fact that they allow for building long and composite search queries. Normally, in studies that involve bibliometric review, one database is used due to the fact that bibliometric indices and literature mapping are difficult to perform on documents retrieved from different databases (Sweileh, 2020). However, it has been shown that using only one database may limit the inclusion of some relevant articles that may be required for analysis on a particular subject matter (Mansoori, 2018; Orimoloye et al., 2020). The use of WOS and Scopus bases will ensure 100% inclusion of PubMed. Therefore, WOS and Scopus are judged to have a comprehensive collection of publications in PubMed and other scientific databases.

### Search Strategy Used for Data Collection

In order for us to create a search query that can recover most of the related amount of research outputs with slightest false-positive outcome, we did a thorough literature review on the subject matter/topic, particularly on studies and systematic reviews to familiarize ourselves with most of the potential keywords related to the search topic (Milan et al., 2013; Mukhopadhyay, 2014; Ramachandraiah et al., 2015; King et al., 2018; Fesseha et al., 2020). The simplest method that was adopted was to use the title/abstract search methodology for keywords related to “meat production” and “nanotechnology.” However, adopting such method will retrieve a large number of documents that may not be needed. Therefore, in order to streamline the title/abstract method that was used, a particular constraint was employed that included the presence of certain “terms” related to meat production or nanotechnology in addition to the title/abstract strategy.

### Search Query of Data Used for the Study

The inclusive search query comprised of precise phrases related to meat production and nanotechnology that were entered into the title/abstract search engine, followed by specific terminologies as a constraint to lessen and remove irrelevant research work that will not contribute to the objective of the present study. The search queries for WOS and Scopus that were used are the following:

**1. Web of Science**

Results: 558

(from Web of Science Core Collection)

You searched for: TITLE: (nano^*^ AND (livestock^*^ OR meat^*^ OR beef^*^ OR mutton^*^ OR pork^*^ OR chevon^*^ OR chicken^*^ OR turkey^*^))

Timespan: 1985–2020. Indexes: SCI-EXPANDED, SSCI, A&HCI, CPCI-S, CPCI-SSH, BKCI-S, BKCI-SSH, ESCI, CCR-EXPANDED, IC.

…Less

Results: 511

(from Web of Science Core Collection)

You searched for: TITLE: (nano^*^ AND (livestock^*^ OR meat^*^ OR beef^*^ OR mutton^*^ OR pork^*^ AND chevon^*^ OR chicken^*^ OR turkey^*^))

Refined by: DOCUMENT TYPES: (ARTICLE)

Timespan: 1985–2020. Indexes: SCI-EXPANDED, SSCI, A&HCI, CPCI-S, CPCI-SSH, BKCI-S, BKCI-SSH, ESCI, CCR-EXPANDED, IC.

…Less

**2. Scopus**

752 document results

TITLE (nano^*^ AND (livestock^*^ OR meat^*^ OR beef^*^ OR mutton^*^ OR pork^*^ OR chevon^*^ OR chicken^*^ OR turkey^*^))

652 document results

TITLE (nano^*^ AND (livestock^*^ OR meat^*^ OR beef^*^ OR mutton^*^ OR pork^*^ OR chevon^*^ OR chicken^*^ OR turkey^*^)) AND (EXCLUDE (PUBYEAR, 2021))

593 document results

TITLE (nano^*^ AND (livestock^*^ OR meat^*^ OR beef^*^ OR mutton^*^ OR pork^*^ OR chevon^*^ OR chicken^*^ OR turkey^*^)) AND (EXCLUDE (PUBYEAR, 2021)) AND (LIMIT-TO (DOCTYPE, “ar”)).

### Analysis and Data Processing

**Figure 1:**
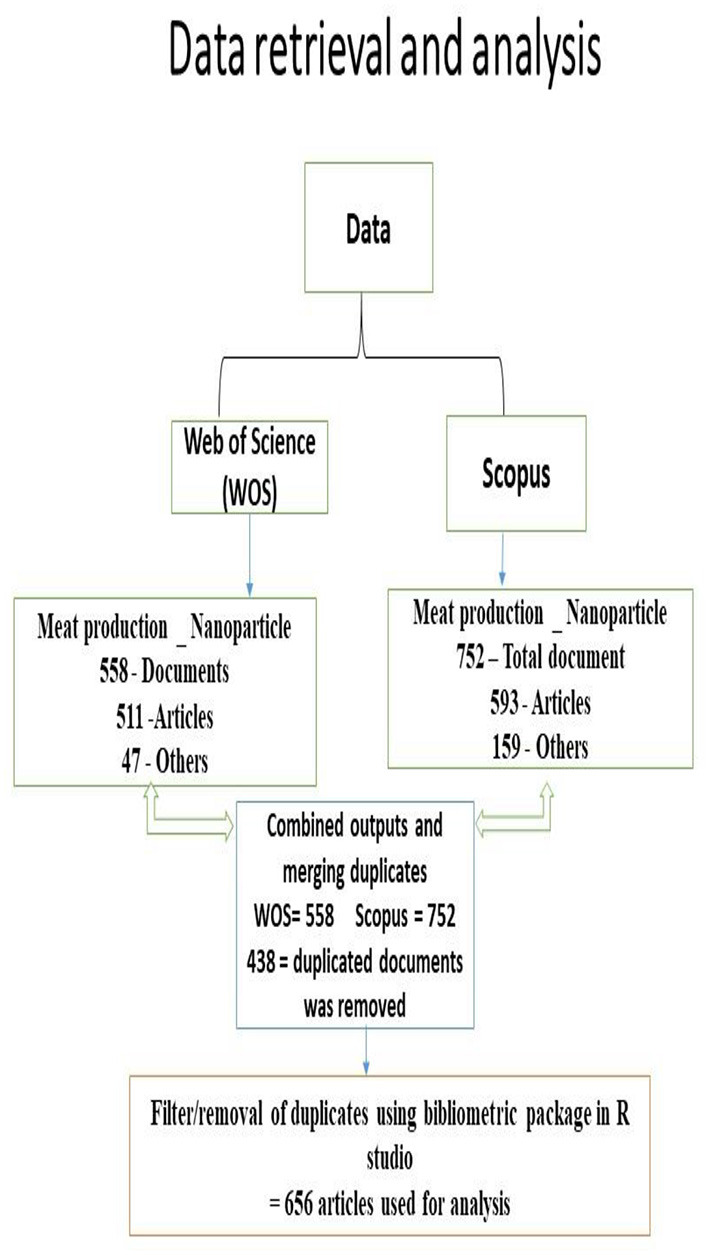
Schematic presentation showing the inclusion and exclusion criteria for outputs selection.

**Figure 2:**
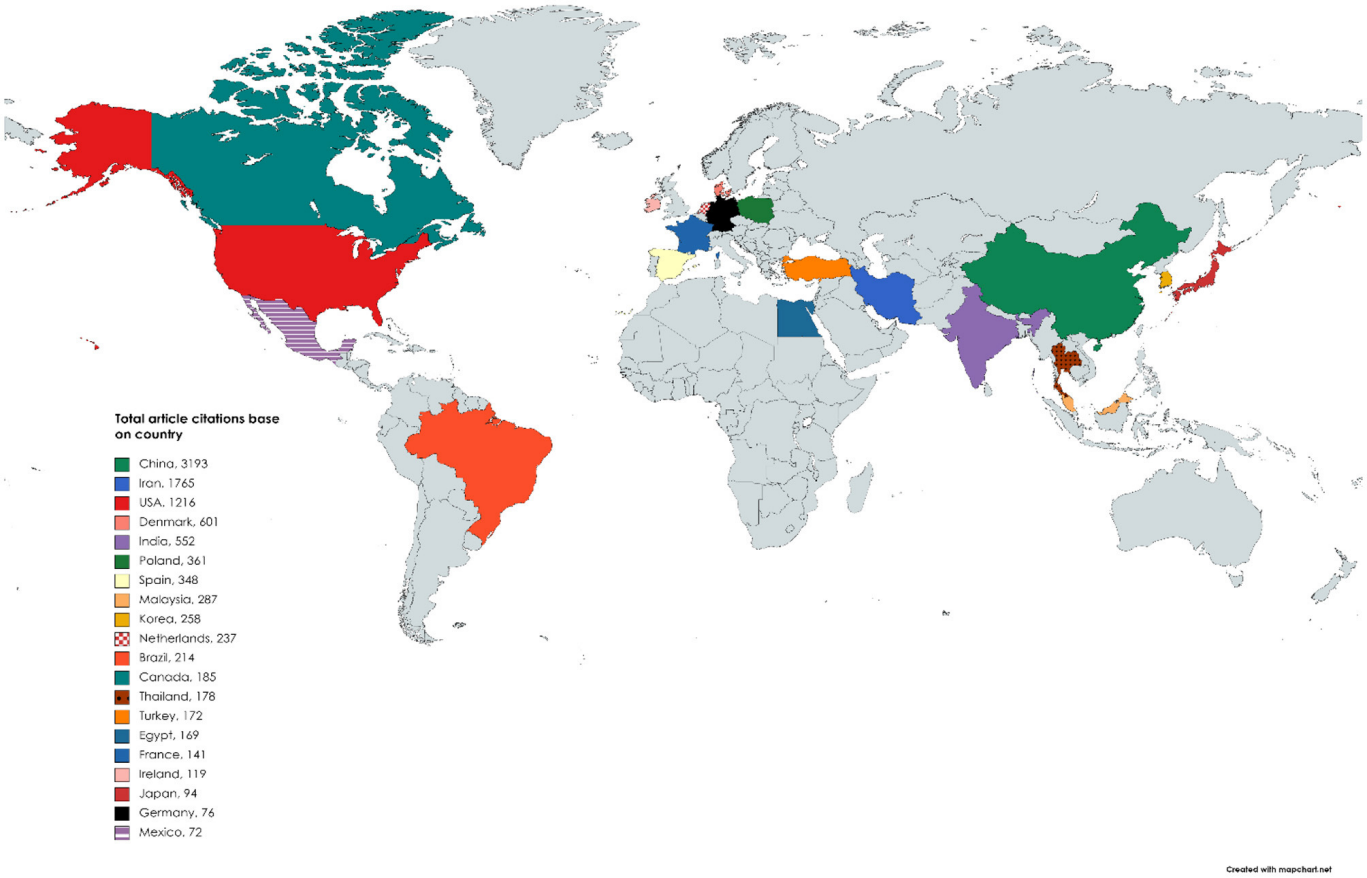
Spatial distribution of top 20 total citations by different countries. Gray color covering depicts the areas that are not part of the top 20 total citations.

The present study analyzed the data retrieved using RStudio v. 4.0.4 software with bibliometrix R-package for bibliometric factors (Aria and Cuccurullo, 2017). All data were transferred into R Studio and refined into a bibliographic data form and structured for duplication (Ekundayo and Okoh, 2018). A schematic diagram of the retrieval and analysis of data can be seen in Figure 1. All duplicated, peer-reviewed articles were restricted to one record in the analysis. In addition, for visualization, the names of authors and author's keywords were extracted. An annual number of articles and total citations (TCs) were also graphed (**Table 2** and Figure 2).

## Results

**Figure 3:**
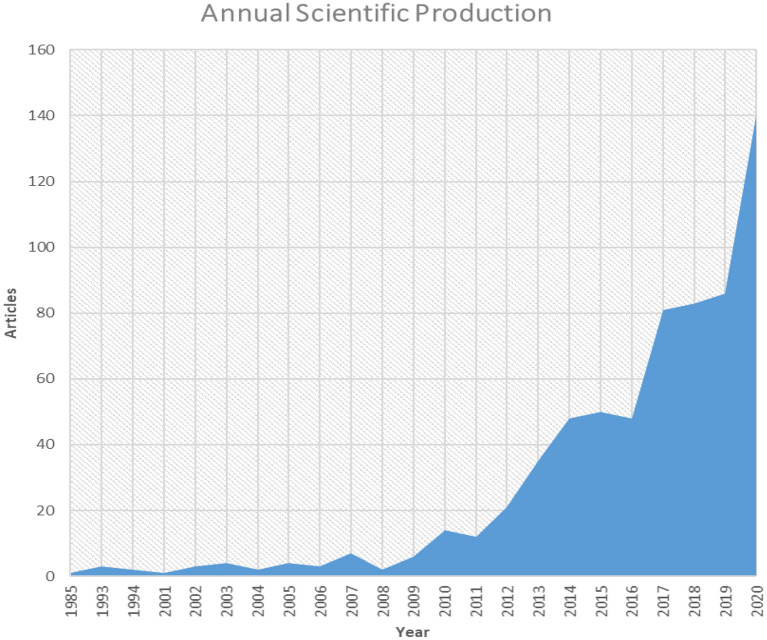
Trend of annual scientific production of research outputs (from 1985 to 2020) in the field of nanotechnology and meat production with an annual growth rate of 25.18%. Meat production studies with the use of nanotechnology show slight fluctuations of research outputs between 1985 and 2008.

From our result, an aggregate of 656 articles were published within the period of analysis; and the analysis characteristics are given in Table 1. The outputs for the surveyed periods comprise of 2,133 authors, with 14 single authors, 0.308 article per author (3.25 authors per article), a collaboration index of 3.31, and a 4.81 coauthors per article. With the exemption of 14 authors who were single authors, all other authors (2,119) had a multiauthor research outputs. An average of 17.02 citations per article was documented during the study period. Likewise, Figure 2 shows research outputs of spatial distribution related to meat production *via* the use of nanotechnology for the top 20 most active countries. China ranked first in the aggregate numbers of articles (*n* = 3,193), followed by Iran (*n* = 1,765), United States of America (USA) (*n* = 1,216), Denmark (*n* =601), and India (*n* = 552), respectively, among others. The result also shows a trend in research outputs that tended toward improving meat production and nanotechnology with an annual growth rate of 25.18% (Figure 3). The research production had some fluctuations between 1985 and 2008 during the survey period; however, it peaked in the subsequent years with an upward trend from 2009 till date (Figure 3). The peak year of article publication in meat production and nanotechnology field was in the year 2020, with an aggregate output of 140 articles (Figure 3). The average article citations (AAC) of most cited countries in the field of meat production and nanotechnology research showed that France (141), Netherlands (118.50), USA (35.76), Spain (34.80), and Denmark (25.04) are leading the chart, respectively (Table 2). The research output related to meat production and nanotechnology for the top 20 most active countries is shown in Table 3. China ranked first in terms of the total sum of articles published (*n* = 140), followed by Iran (*n* = 111) and India (*n* = 37). The frequency of research outputs varied among the top 20 countries from 0.00957 to 0.25510. Furthermore, the top nations with multiple country publications (MCP) were China and Netherlands, which tied in the first position (23), followed by Iran (11) in the second position, and USA (10) in the third position, respectively, among others. While the nations ranked in top positions for single country publications of research outputs are China (137), Iran (100), and India (34) among others (Table 3). Among the top 20 most frequently used keywords by researchers in the field of meat production *via* the use of nanotechnology techniques, nanoparticles (75; 11.43%) was ranked first, followed by meat (62; 9.45%), chitosan (57; 8.68%), performance (53; 8.07%), and animals (49; 7.46%) among other keywords used by authors (Table 4).

**Table 1:** General information on the retrieved published documents on meat production and nanotechnology from WOS and Scopus data base.

**Description**	**Results**
**Main information about data**	
Timespan	1985:2020
Sources (journals, books, etc.)	321
Documents	656
Average years from publication	4.83
Average citations per documents	17.02
Average citations per year per doc	3.028
References	23,290
**Document types**	
Article	641
Article; book chapter	4
Article; proceedings paper	11
**Document contents**	
Keywords plus (ID)	3,080
Author's keywords (DE)	1,848
**Authors**	
Authors	2,133
Author appearances	3,157
Authors of single-authored documents	14
Authors of multi-authored documents	2,119
**Authors collaboration**	
Single-authored documents	16
Documents per author	0.308
Authors per document	3.25
Coauthors per documents	4.81
Collaboration index	3.31

**Table 2:** The top 20 most cited countries in terms of average article citations (AAC) in the field of meat production and nanotechnology from 1985 to 2020.

**S/N**	**Country**	**Total citations**	**Average article citations (AAC)**
1	China	3,193	19.96
2	Iran	1,765	15.90
3	USA	1,216	35.76
4	Denmark	601	25.04
5	India	552	14.92
6	Poland	361	9.76
7	Spain	348	34.80
8	Malaysia	287	19.13
9	Korea	258	13.58
10	Netherlands	237	118.50
11	Brazil	214	26.75
12	Canada	185	12.33
13	Thailand	178	22.25
14	Turkey	172	10.12
15	Egypt	169	8.05
16	France	141	141.00
17	Ireland	119	19.83
18	Japan	94	11.75
19	Germany	76	7.60
20	Mexico	72	12.00

**Table 3:** The top 20 publications by countries in the field of nanotechnology and meat production research.

**S/N**	**Country**	**Articles**	**Rank**	**Frequency**	**SCP**	**MCP**	**MCP_Ratio**
1	China	160	1	0.25510	137	23	0.1437
2	Iran	111	2	0.17703	100	11	0.0991
3	India	37	3	0.05901	34	3	0.0811
4	Poland	37	3	0.05901	32	5	0.1351
5	USA	34	4	0.05423	24	10	0.2941
6	Denmark	24	5	0.03828	1	23	0.9583
7	Egypt	21	6	0.03349	18	3	0.1429
8	Korea	19	7	0.03030	17	2	0.1053
9	Turkey	17	8	0.02711	16	1	0.0588
10	Canada	15	9	0.02392	11	4	0.2667
11	Malaysia	15	9	0.02392	9	6	0.4000
12	Indonesia	12	10	0.01914	12	0	0
13	Germany	10	11	0.01595	9	1	0.1000
14	Spain	10	11	0.01595	9	1	0.1000
15	Brazil	8	12	0.01276	8	0	0
16	Japan	8	12	0.01276	6	2	0.2500
17	Thailand	8	12	0.01276	6	2	0.2500
18	Russia	7	13	0.01116	7	0	0
19	Ireland	6	14	0.00957	0	6	1
20	Mexico	6	14	0.00957	4	2	0.3333

*SCP, Single Country Publications; MCP, Multiple Country Publications*.

**Table 4:** Top 20 most frequently used words by researchers in the field of meat production through nanotechnology.

**S/N**	**Key words**	**Rank**	**Frequency**	**% of 656**
1	Nanoparticles	1	75	11.43
2	Meat	2	62	9.45
3	Chitosan	3	57	8.68
4	Performance	4	53	8.07
5	Animals	5	49	7.46
6	Article	6	39	5.94
7	Animal	7	37	5.64
8	Growth	8	33	5.03
9	Quality	8	33	5.03
10	Toxicity	8	33	5.03
11	Chemistry	9	30	4.57
12	Growth-performance	10	29	4.42
13	Listeria-monocytogenes	10	29	4.42
14	Shelf-life	10	29	4.42
15	Meats	11	26	3.96
16	Adsorption	12	25	3.81
17	Clenbuterol	12	25	3.81
18	Non-human	12	25	3.81
19	Antibacterial activity	13	24	3.65
20	Procedures	13	24	3.55

*NB: % of 656 = total sum of articles published on nanotechnology and meat production and from 1985 to 2020*.

**Figure 4:**
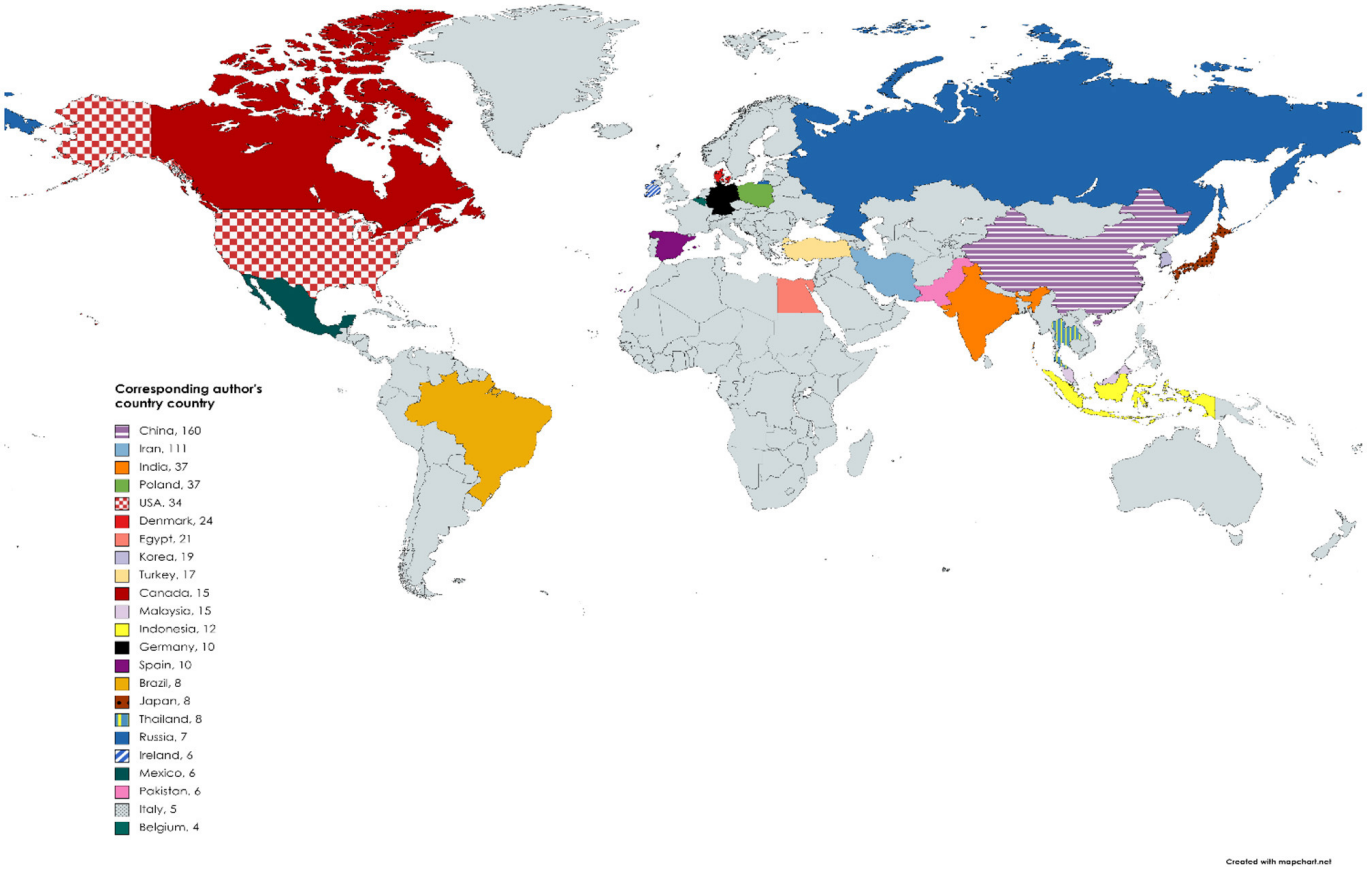
Spatial mapping of the top 20 most productive countries based on the number of research articles on nanotechnology and meat production (Corresponding author's countries). Gray color covering depicts the areas that are not part of the top 20 countries.

**Figure 5:**
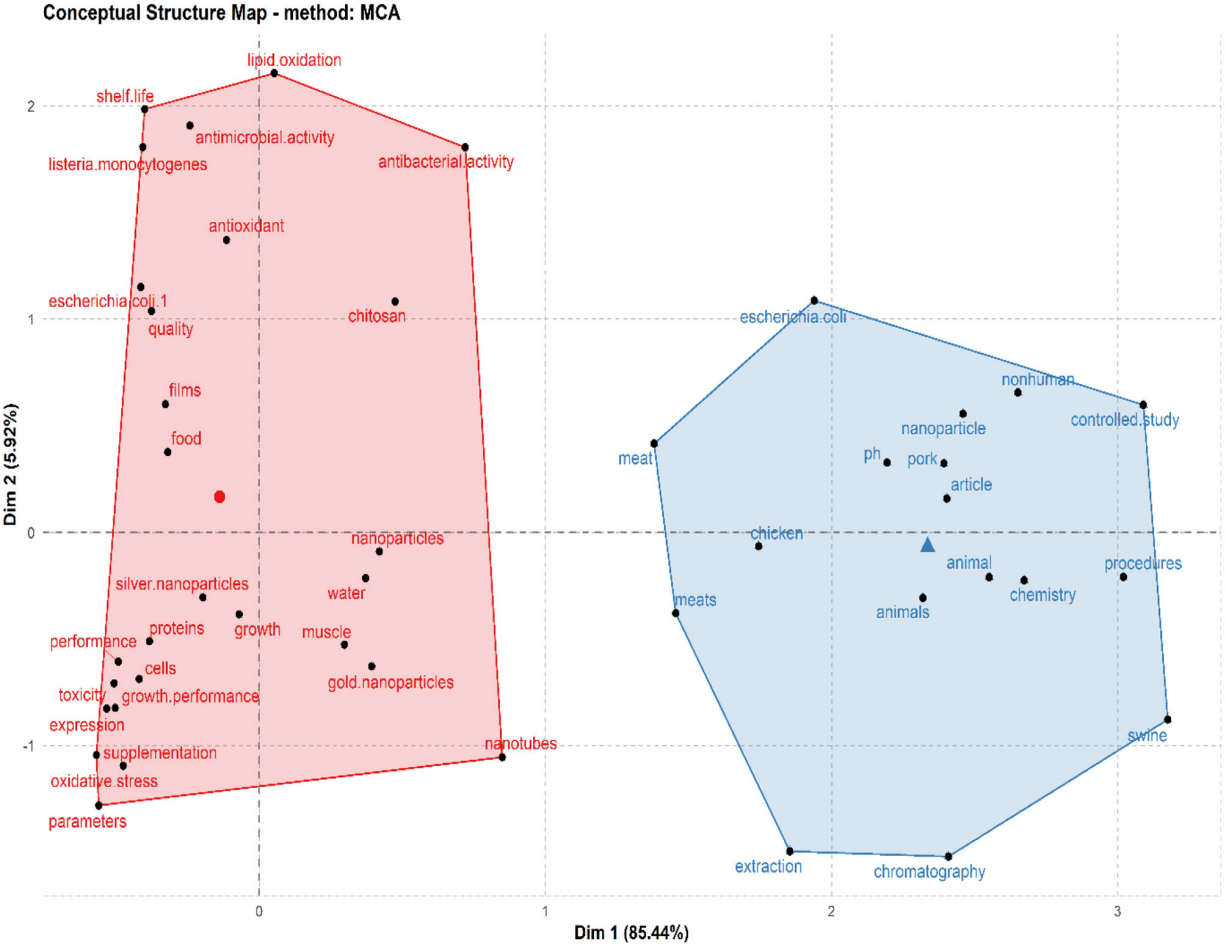
Common conceptual frames related to meat production *via* the use of nanotechnology research studies. The 656 retrieved articles showed K-means clustering with two (2) clusters reflecting models of nanomaterials (nanotubes, silver nanoparticles, and gold nanoparticles) for improvement of meat (chicken and pork), meat qualities (ph, shelf-life, lipid oxidation, antioxidant, etc.), performance (growth, oxidative stress, muscle meat), and healthy meat (antibacterial activities e.g., listeria, *Escherichia coli*) commonly linked to nanotechnology and meat production.

Table 5 shows the top 20 most relevant/productive authors in the field of meat production and nanotechnology. E. Sawosz ranked first; coauthoring 28 (4.27%) articles, A. Chwalibog was ranked in the second position with 22 (3.35%) articles, while K. Ognik maintained the third position with 18 (2.74%) articles. The h_index (based on TCs) was 16 (TC = 486) for E. Sawosz, 13 (TC = 384) for A. Chwalibog, and 10 (TC = 403) for L. Lin who ranked first, second, and third positions, respectively (Table 5). Countries from Asia including China (160), Iran (111), and India (37) contributed the highest number (based on corresponding authors' countries) of published items to meat production and nanotechnology, with Europe (Poland: 37) and America (USA: 34) following suit in that order (Figure 4). The shared conceptual frames in retrieved publications as was explained by K-means clustering with two (2) clusters of 5.92 and 85.44% elements showed research responses focused on models of nanomaterials (nanotubes, silver nanoparticles, and gold nanoparticles) for improvement of meat (chicken and pork), meat qualities (ph, shelf life, lipid oxidation, antioxidant, etc.), livestock performance (growth, oxidative stress, muscle meat), and healthy meat (antibacterial activities e.g., listeria, *Escherichia coli*) commonly linked to meat production through nanotechnology (Figure 5). Table 6 shows the top 20 most cited articles on meat production and nanotechnology with their Digital Object Identifier (DOI) given as 10.1016/j.chroma.2006.06.024 (by G.Z. Fang, 2006; TC: 183); 10.1016/j.foodcont.2017.08.015 (by S. Noori, 2018; TC: 154); and 10.4315/0362-028X-68.9.1804 (by M. Varshney, 2005: TC: 149), among others.

**Table 5:** Top 20 relevant/productive authors on meat production through nanotechnology.

**S/N**	**Author**	**Rank**	**H_index**	**G_index**	**M_index**	**TC**	**NP**	**% of 656**	**PY_start**
1	Sawosz E	1	16	21	1	486	28	4.27	2006
2	Chwalibog A	2	13	19	1	384	22	3.35	2009
3	Ognik K	3	7	12	1.16	163	18	2.74	2016
4	Lin L	4	10	13	2	403	13	1.98	2017
5	Li Y	5	7	11	0.41	472	11	1.67	2005
6	Cui H	5	9	11	1.80	301	11	1.67	2017
7	Grodzik M	5	9	11	0.56	246	11	1.67	2006
8	Stepniowska A	5	5	9	0.83	90	11	1.67	2016
9	Wang X	6	6	10	0.85	191	10	1.52	2015
10	Chen S	7	8	9	0.88	169	9	1.37	2013
11	Wang J	7	5	8	0.41	80	9	1.37	2010
12	Wang Y	7	7	9	0.63	232	9	1.37	2011
13	Jankowski J	8	4	5	1	34	8	1.21	2018
14	Jaworski S	8	7	8	0.77	157	8	1.21	2013
15	Li H	8	5	8	0.55	142	8	1.21	2013
16	Sembratowicz I	8	3	7	0.50	58	8	1.21	2016
17	Zhang H	8	6	8	0.35	228	8	1.21	2005
18	Hotowy A	9	6	7	0.50	119	7	1.06	2010
19	Kozlowski K	9	4	7	0.80	54	7	1.06	2017
20	Li J	9	6	7	0.54	127	7	1.06	2011

*NB: % of 656 = total sum of articles published on nanotechnology and meat production between 1985 and 2020; NP, Number of Publications; PY_start, Publication year start*.

**Table 6:** Top 20 most cited articles on meat production and nanotechnology.

**S/N**	**Paper**	**Journal name**	**Digital object identifier (DOI)**	**Total citations (TC)**	**Rank**	**TC per year**	**Normalized TC**
1	Fang GZ, 2006	Journal of Chromatography A	10.1016/j.chroma.2006.06.024	183	1	11.43	1.8179
2	Noori S, 2018	Food Control	10.1016/j.foodcont.2017.08.015	154	2	38.50	9.7946
3	Varshney M, 2005	Journal of Food Protection	10.4315/0362-028X-68.9.1804	149	3	8.76	2.0694
4	Dehnad D, 2014	Carbohydrate Polymers	10.1016/j.carbpol.2014.03.063	143	4	17.87	5.1416
5	Lavial F, 2007,	Development	10.1242/dev.006569	141	5	9.40	2.964
6	Nasrollahzadeh M, 2016	Applied Catalysis B: Environmental	10.1016/j.apcatb.2016.02.042	135	6	22.5	7.4568
7	Varshney M, 2005	Journal of Food Protection	10.4315/0362-028X-68.9.1804	133	7	7.82	1.8472
8	Loeschner K, 2013	Analytical and Bioanalytical Chemistry	10.1007/s00216-013-7228-z	131	8	14.55	4.2572
9	Zhang KL, 2002	Analytical Biochemistry	10.1006/abio.2002.5719	124	9	6.20	2.3544
10	Peters RJB, 2014	Analytical and Bioanalytical Chemistry	10.1007/s00216-013-7571-0	121	10	15.12	4.3506
11	Peters RJB, 2014	Analytical and Bioanalytical Chemistry	10.1007/s00216-013-7571-0	116	11	14.50	4.1708
12	Cai SJ 2012	Poultry Science	10.3382/ps.2012-02160	106	12	10.60	2.8106
13	Akbar A, 2014	Food Control	10.1016/j.foodcont.2013.09.065	99	13	12.37	3.5596
14	Liu SF, 2015	Angewandte Chemie International Edition	10.1002/anie.201501434	92	14	13.14	4.2125
15	Hu CH, 2012	Animal Feed Science and Technology	10.1016/j.anifeedsci.2012.08.010	86	15	8.60	2.2803
16	Morsy MK 2014	Journal of Food Science	10.1111/1750-3841.12400	81	16	10.12	2.9124
17	Hu J, 2015	LWT- Food Science and Technology	10.1016/j.lwt.2015.03.049	81	16	11.57	3.7088
18	Huang W, 2012	Food Research International	10.1016/j.foodres.2012.06.026	81	16	8.10	2.1477
19	Panea B, 2014	Journal of Food Engineering	10.1016/j.jfoodeng.2013.09.029	80	17	10	2.8764
20	Moazzen M, 2013	Talanta	10.1016/j.talanta.2013.07.005	79	18	8.77	2.5673

**Figure 6:**
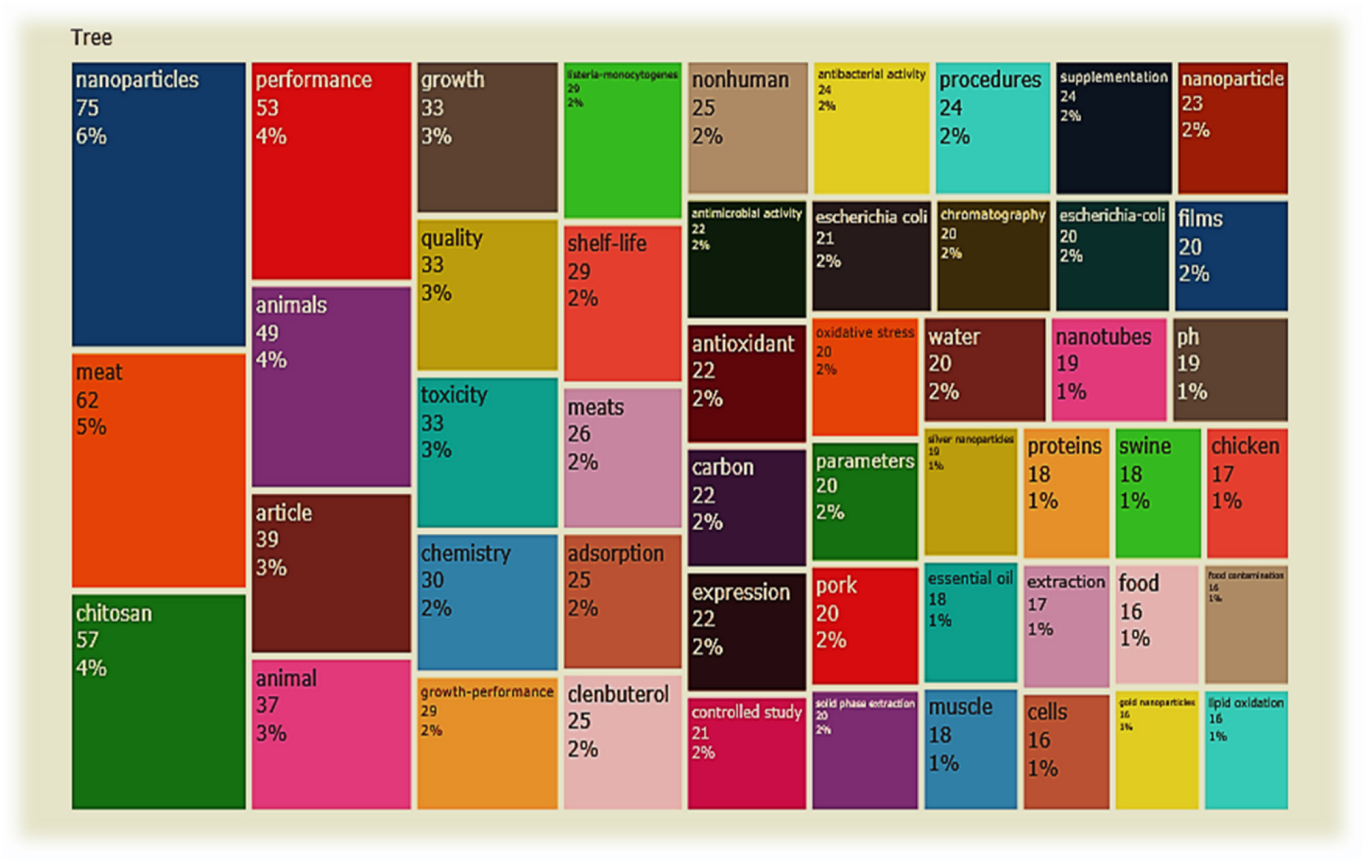
Tree map of discipline distribution in the field of meat production and nanotechnology research.

**Figure 7:**
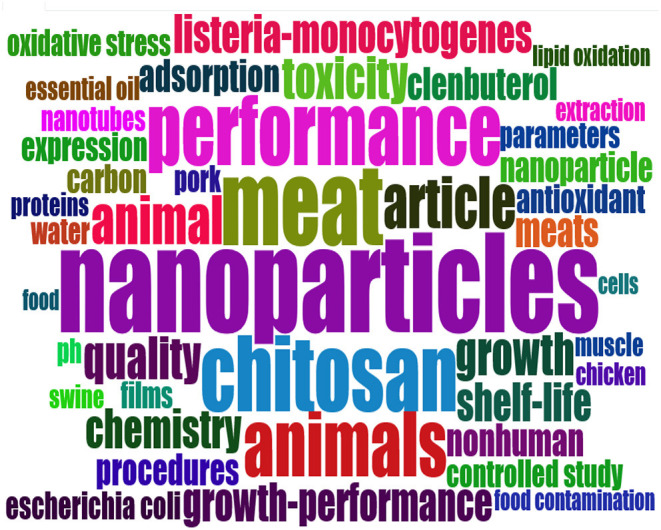
Word cloud on nanotechnology and meat production studies.

**Figure 8:**
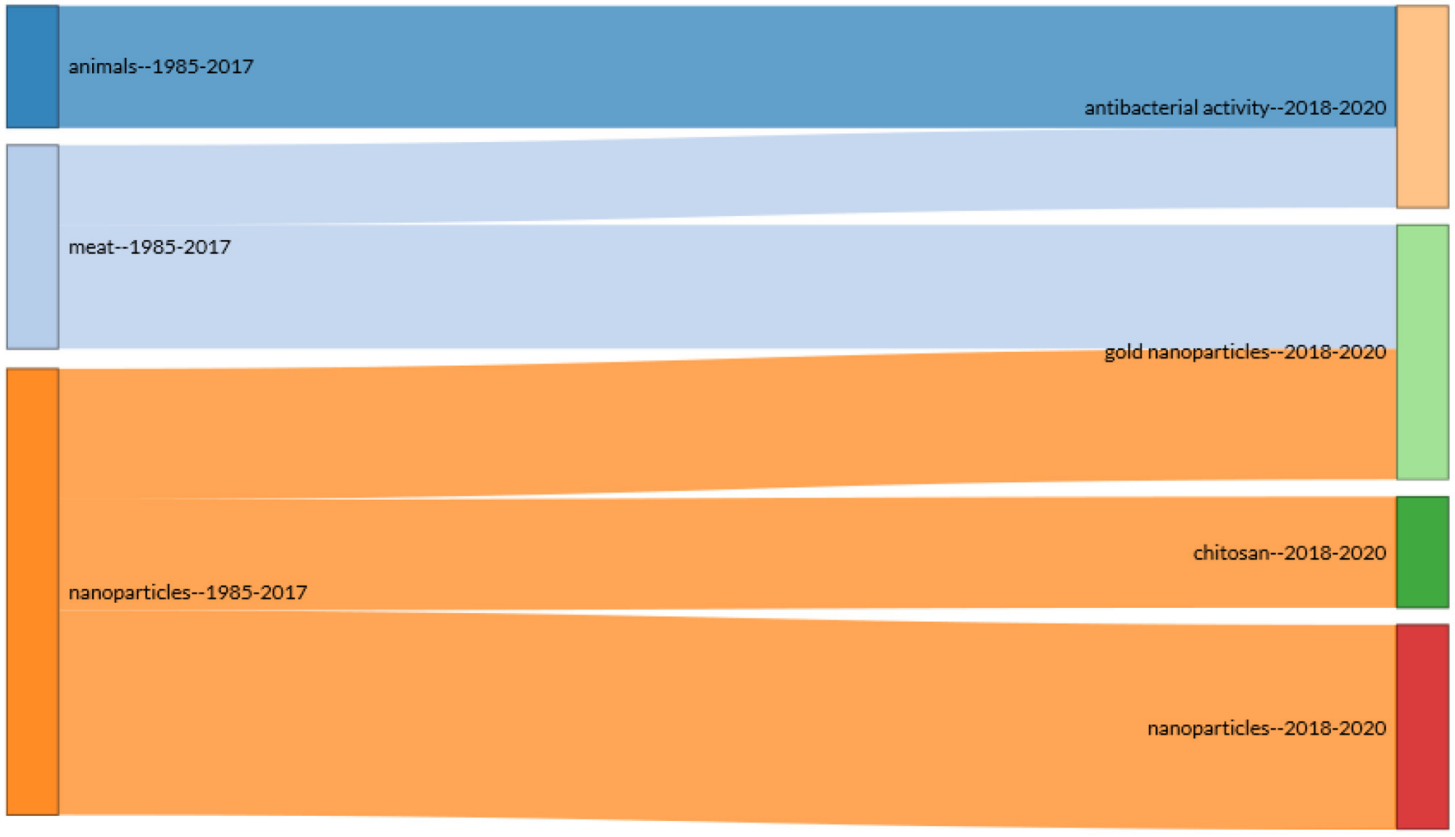
Thematic evolution of author keywords on nanotechnology and meat production research from 1985 to 2020.

The top 20 journals with the most published articles in the field of meat production and nanotechnology are listed in Table 7. These journals address a range of areas including nanomaterials (nanotubes, silver nanoparticles, and gold nanoparticles), livestock (chicken and pork), meat qualities (ph, shelf life, lipid oxidation, antioxidant, etc.), livestock performance (growth, oxidative stress, muscle meat), and health (antibacterial activities e.g., listeria, *Escherichia coli*), among others. Food Chemistry, International Journal of Biological Macromolecules, Lwt-Food Science and Technology, and Food Packaging and Shelf Life reflect active areas in meat production and nanotechnology research. Food Chemistry journal ranked first (*n* = 22 articles, 3.35%), followed by International Journal of Biological Macromolecules and Lwt-Food Science and Technology (*n* = 16 articles, 2.43%) with both tied in the second position, and followed by Food Packaging and Shelf Life Journal (*n* = 15 articles, 2.28%) in the third position (Table 7). The statistical analysis of the articles linked to the production of quality meat through the use of nanotechnology revealed that we can infer that meat produced from livestock farming involves a number of research directions, including Animal Husbandry, Food Processing, Food Packaging, Microbiology, Food Science and Technology, Chemistry, Botany, Food Engineering, etc. The present subject matter presents a good drive of advancement and a large space for research in the field. Some of the inferred identified areas are in Chemistry, Microbiology, Botany, Food Processing, and Packaging, among others (Figure 6). The information in Figure 7 shows the result of the word cloud of commonly occurring keywords in meat production and nanotechnology studies. It is worthy to note that each keyword size, as seen in the word cloud network (Figure 7), suggests its strength and occurrence in the literatures related to meat production and nanotechnology research outputs. It can also be inferred that the closer the keywords to each other in the word cloud, the more likely their interrelation in the literature during the study period. The word cloud easily visualizes the popular words in meat production and nanotechnology research, which makes it stress-free to recognize the areas of concentration in this niche area. Figure 8 presents the results of the evaluated thematic progress and the selected research clusters and origin, based on the incidence of key terms in meat production and nanotechnology published articles. The thematic evolution epitomizes how key themes surfaced over time in the designated authors' keywords. The result from Figure 8 depicts that the steady themes used by authors from 1985 to 2017 are animals, meat, and nanoparticles and they metamorphosed to antibacterial activity, gold nanoparticles, chitosan, and nanoparticles from 2018 to 2020.

**Table 7:** The top 20 articles that are relevant in the field of meat production and nanotechnology from 1985 to 2020.

**S/N**	**Journal**	**Rank**	**Frequency**	**% of 656**	**Cum Freq**
1	Food Chemistry	1	22	3.35	22
2	International Journal of Biological Macromolecules	2	16	2.43	38
3	Lwt-Food Science and Technology	2	16	2.43	54
4	Food Packaging and Shelf life	3	15	2.28	69
5	Poultry Science	4	10	1.52	79
6	Scientific Reports	4	10	1.52	89
7	Analytical and Bioanalytical Chemistry	5	9	1.37	98
8	Archives of Animal Nutrition	5	9	1.37	107
9	Meat Science	6	8	1.21	115
10	PLoS ONE	6	8	1.21	123
11	Food Analytical Methods	7	7	1.07	130
12	Food Control	7	7	1.07	137
13	Iranian Journal of Applied Animal Science	7	7	1.07	144
14	Journal of Chromatography A	7	7	1.07	151
15	Annals of Animal Science	8	6	0.91	157
16	Environmental Science and Pollution Research	8	6	0.91	163
17	Food Hydrocolloids	8	6	0.91	169
18	International Journal of Food Microbiology	8	6	0.91	175
19	Livestock Science	8	6	0.91	181
20	Plant Archives	8	6	0.91	187

*NB: % of 656 = total sum of articles published on meat production and nanotechnology between 1985 and 2020*.

## Discussions

The present scientometric analysis of meat production and processing *via* the use of nanotechnology examined global research trends between 1985 and 2020 based on data retrieved from WoS and Scopus. We found that the number of research articles on meat production and processing through nanotechnology increased non-linearly from one (1) article in 1985 to 656 articles in 2020. However, there was a slow trend in the rate of increase, which was noticed between the year 2003 and 2009, as there were fluctuations in research outputs on the use of nanotechnology to advance meat production. Conversely, there was a steady pickup in the rate of outputs in this field from 2010 (*n* = 14) to 2020 (*n* = 140) suggesting that research on the use of nanotechnology application for meat production has been of broad interest in the past 15 years. This likely may be due to the continual exploration of nanotechnology-related research in advancing meat production by several authors from different parts of the world, especially in Asia, Europe, and America (Hu et al., 2015; Shavisi et al., 2017; Noori et al., 2018; Ognik et al., 2018).

Furthermore, from the annual scientific production graph in Figure 3 (with annual increase of 25.18%), it indicates that scientific outputs on meat production and processing through the use of nanotechnology are growing rapidly, and it suggests that it will further increase in the future. Although, the use of nanotechnology in the production of meat and in agriculture has not been well-exploited when compared to other fields, especially in medicine (Nikalje, 2015; Ibrahim, 2020). Its application in the field of medicine has led to novel and innovative methods in several areas of medicine (Fesseha et al., 2020), from which scientist in the field of agriculture and animal production can learn a leave from. With the current trend of food challenges and food insecurity in most countries, this may further drive the present generation of new scientific knowledge toward a significant rise in the number of researches to be done on this subject matter.

As it has been commonly observed with other research fields, a great number of the foremost authors championing the application of nanotechnology in meat production researches were mostly from developed countries like China, USA, Denmark, and Poland, with few from low-income countries, thus following the related trend of low efficiency of the regions in several other research areas. Some authors are of the opinion that the economic prowess (growth) of a certain country stimulates their research priority and productivity (Zhang et al., 2010; Peng et al., 2015). The situation of food insecurity and shortage of food commonly experienced in developing countries, and mostly in countries from sub-Saharan Africa, should inspire researchers in these countries to explore the possibilities of carrying out more studies in the use of nanotechnology to advance meat production. According to Fesseha et al. (2020), several devices of nanotechnology including nanomaterials, microfluidics, nanosensors, and bioanalytical have been used to improve various conditions linked with animal production, health, reproduction, and prevention of diseases in livestock. These nanotechnology principles may be used to improve meat production in these countries that are yet to adopt the techniques.

The result from the present study, as seen in Table 6, further showed that some of the most frequently cited articles were studies related to employing the use of nanoparticles to improve meat quality and shelf life and as an antimicrobial agent to mitigate against the growth of harmful bacteria that may cause meat spoilage and poisoning. For instance, the efforts by some authors to:

(a). Produce a nanoemulsion-based edible coating containing ginger (*Zingiber officinale*) essential oil to improve both the safety and quality attributes of chicken (article by Noori et al., 2018).

(b). Use magnetic nanoparticle-antibody conjugates for the separation of *Escherichia coli* O157:H7 in ground meat (article by Varshney et al., 2005).

(c). The efficacy of nanocellulose films for extending the shelf life of ground meat (Dehnad et al., 2014) are among other excellent articles that were frequently cited by other researchers.

Other authors focused on the use of nanoparticles (e.g., nano-selenium) as supplement in poultry feed to boost meat quality, oxidation resistance, immune function, and muscle size (article by Cai et al., 2012). These novel research outputs are a pointer to the relevance of nanotechnology in advancing meat production. According to Singh et al. (2016), nanotechnology has a unique prospect to give benefits to the meat sector in the complete cycle of meat production from the improvement of meat texture, odor, and taste to the production of meat with low fats *via* the use of advanced packaging materials (coated with nanoparticles) and ensuring the safety of products by employing bio-innovative techniques (such as biosensors).

China and Iran dominated the ranks of top 20 countries that are furthermost in active research in the application of nanotechnology for meat production in terms of numbers of articles and citations (Tables 2, 3). One major reason for any country to fall into the category of more number of article production and citations in a given field may be attributed to its economic prowess/strength and accessibility to research facilities and funding (Liu et al., 2013; Peng et al., 2015; Zyoud, 2017). In addition, the increased productivity in this field could be ascribed to a possible high-level involvement in both local and multinational collaborations with other research establishments, which are potential boosters that increase impact research visibility and citation frequency (Liu et al., 2013; Zyoud, 2017). Although, the United States has shown dominance in several other research areas (Bundschuh et al., 2015; Geaney et al., 2015; Bruggmann et al., 2017), in the present study, it was ranked in the fifth position in the list of top 20 most published articles on nanotechnology and meat production (Table 3). Another factor that can influence multinational networks of a country can be linked to the authors' multiple affiliations (Ekundayo and Okoh, 2018). However, the relatively low contributions to research on nanotechnology and meat production as reflected from the present study by developing countries including countries from Africa (with only Egypt making the list in the top 20 countries) may not be unrelated to the fact that most researches done in these places are usually self-funded or independent studies that do not attract research funding from their government or non-governmental organizations (NGOs).

The 20 topmost countries with multiple collaboration (MC) on nanotechnology and meat production studies showed collaboration allied was mostly among authors from developed and high financially stable countries including China and the United States (Table 3). This observation is similar to the trends of publications reported in collaboration network of countries in many other research fields (Orimoloye and Ololade, 2021; Smith et al., 2021). A common trend on bibliometric studies shows that alliances between developed and developing countries are scarce in several scientific fields (Ekundayo and Okoh, 2018; Orimoloye and Ololade, 2021). From the present study, it was observed that, among authors in China, collaboration pathways were largely local, as suggested by a large number of single publications (SCP; *n* = 137), but with only 23 MCP. On the other hand, collaborations by authors in Denmark (SCP; *n* = 1, MCP; *n* = 23) and Ireland (SCP; *n* = 0; MCP; *n* = 6) tended to be multinational, which is more appreciated because of the need for diffusion of innovative ideas from highly productive countries in the field of nanotechnology and meat production research to the emerging countries in the field. The absence of collaboration network in countries like Indonesia, Brazil, and Russia may be linked to the low number of research outputs (SCP; *n* = 12, 8, and 7) from these countries, respectively (Table 3). Collaboration in scientific research from both intra- and international institutions between developing and developed countries could afford a more robust opportunity for pulling resources (funds and facilities) and more man power for division of labor to tackle the important research gaps in the field of nanotechnology and meat production.

Furthermore, it was observed from the present study that there was a swing in the rankings among the top 20 countries that are most active in the field of nanotechnology and meat production research when productivity was evaluated using the criteria of TC per country (Figure 2 and Table 2). Similar trend was also observed in another study (Ekundayo and Okoh, 2018). The indication with this kind of swing in rankings when using the total number of citations to judge an author or country's outputs may show its unreliability as an accurate yardstick for productivity. According to Fricke et al. (2013), the rate of citation does not really reflect the publication outputs of an author or a country. This is because the smaller the number of articles used for estimation, the greater the impact of a few regularly cited articles (Fricke et al., 2013). Most researchers have been observed to engage in self-citations, while others give inaccurate citations when writing their manuscripts, which in turn may produce false quality and quantitative metrics of TCs about a particular author or country.

The most regularly revealed keywords and research disciplines (including article outlets) associated with nanotechnology and meat production studies mirrored the research hotspot during the survey period, which include among others nanomaterials (nanotubes, silver nanoparticles, and gold nanoparticles); meat (chicken and pork); meat qualities (ph, shelf life, lipid oxidation, antioxidant etc.); livestock performance (growth, oxidative stress, muscle meat); and antibacterial activities (against listeria, *Escherichia coli*, etc.). This finding revealed some of the successful research work related to the application of nanotechnology in advancing meat production, meat processing, and packaging and some correlated functions of nanomaterials to mitigate against meat spoilage and poisoning. Likewise, the keywords and research discipline revealed from our study points out some of the efforts made by authors to promote research work on the application of current nanotechnology techniques used in meat production in order to gain an understanding of the future prospect of these techniques to the meat industry and in extension to livestock farming as a whole. These findings (most regularly revealed keywords) were supported by other conceptual framework indicators such as tree map and word cloud (Figures 6, 7).

Important to note that, one of the top 20 most cited articles by Moazzen et al. (2013), in their study, pointed out the effectiveness of using magnetic carbon nanotubes to detect carcinogenic compounds (polycyclic aromatic hydrocarbons) in grilled meat samples (Table 6). This nanotechnology technique has long being developed. The novel study by Moazzen et al. (2013) is an important aspect (among other novel research work) that revealed the innovative use of nanotechnology in the prevention of possible health problems (cancer) that may result in the consumption of meat by humans. However, we noticed from our study that most of the work done with nanotechnology on meat production and its research directions (word clouds, tree map, and conceptual structure map) were carried out on chicken and pork (Figures 5–7) with very limited studies, if any, on other meat products (such as beef, mutton, chevon, etc.). More research on this subject matter should however, be tailored toward this direction as these livestock (cattle, goat, sheep, etc.) play a significant role in the production of meat and its products worldwide. A scientometric analysis accompanied with a meta-analysis or a narrative review in nanotechnology and meat production research may also be of benefit to the pool of knowledge in this area.

The information in Figure 8 showed the surveyed thematic progression and the recognized research groups, based on the incidence of vital terms in nanotechnology and meat production published research outputs. The thematic evolution depicts how terms or key themes surfaced over the surveyed period (1985–2020) in the selected authors' keywords. The entire research process in the area of nanotechnology and meat production can be comprehended by means of an all-inclusive observation of the tree maps, emergent keyword maps, and thematic evolutionary paths. The themes used by authors, from our observation from 1985 to 2017, are animals, meat, and nanoparticles, and later they metamorphosed to antibacterial activity, gold nanoparticles, and chitosan from 2018 to 2020; these have remained the themes till date. This progression in the thematic evolution of keywords indicates the efforts researchers had put in place to advance knowledge in meat production *via* the use of nanotechnology (gold nanoparticles).

Furthermore, our observations visibly show that the core theme of the research revolves around the exploration of the drivers of novel techniques to boost meat production and the search for solutions to reduce the problems of health risk during processing and packaging of meat for human consumption (Allan et al., 2021). Meat, like all other food and nutrition issues, has received global attention, particularly in developing countries such as Africa, parts of Asia, and Latin America, where food and nutrition security is a major concern. According to Singh et al. (2016) and Nile et al. (2020), the key areas of nanotechnology application to meat production include improving processing, pathogen detection, flavor and nutrition, delivery methods, functionality of muscle foods, and the cost-effectiveness of storage (in terms of shelf life) and distribution.

Over the years, there has been a novel advancement in the meat production procedures (processing, packaging, and preservation—shelf life) both at the local and industrial level with the aim to obtain meat products with better attributes (Singh et al., 2016). One of the driving forces behind the implementation of newer techniques in boosting meat production is clearly the profitability in the industry, which is also directly linked to the consumer acceptance of meat and its products. The consumer acceptance of any product has been shown to depend on the value-added benefits in terms of nutritional value, safety, shelf life, taste, texture etc. (Singh et al., 2016). Beyond reasonable doubt, this new field of nanotechnology research is hoped to reform the meat industry and in extension the livestock sector by improving production systems if harnessed judiciously (Singh et al., 2016).

For the sake of consumer (s) safety, it is wise to have an all-inclusive information with respect to the interface between nanoparticles and the human body system (cells, tissues, and organisms), particularly in relation to potential hazards to human health (Devasahayam, 2017). For example, it has been reported that nanotechnology has a huge impact on food during processing and packaging (Chaudhry and Castle, 2011). According to Maisanaba et al. (2015), some nanoparticles used in food packaging may penetrate into the human body *via* several ways such as inhalation, ingestion, or cutaneous exposure. As soon as these nanoparticles enter into the human system, they will no doubt come into contact with a vast variety of biomolecules (lipids, sugars, and proteins), which in turn will dissolve in the human body fluids including interstitial fluid between cells, lymph, or blood (Farhoodi, 2016). Research works on silver nanoparticles and titania showed that these nanomaterials possess the potential to enter the blood circulation and their insolubility leads to accumulation in organs of human body (Carrero-Sánchez et al., 2006; Rhim et al., 2013). According to Dimitrijevic et al. (2015), the two known organs that are solely responsible for the carrying of nanoparticles, mediating their passage from intestine to the blood circulation, are sleep and liver. Very scanty research have observed the potential health hazard of nanoparticles resulting from food processing and packaging, besides the information that relates to their bioavailability, biodistribution, and their transportation in the human body system. During consumption of food, certain nanoparticles may inadvertently come in contact with the gut system (GIT) through migration or leaching of these nanoparticles during nanopackaging to food commodities (Szakal et al., 2014; He and Hwang, 2016). This unintended transportation/diffusion of unwelcomed packaging components into food final products such as meat may raise the safety fears of consumers.

There is a possibility that some nanoparticles could be carcinogenic or toxic in nature. However, from the studies discussed in the present paper, there was no instance of carcinogenic incidence that was reported from the use of nanoparticles to boost meat production. Notwithstanding, concerns about a potential migration of nanoparticles (which may be harmful for human consumption) during processing of meat (such as packaging) to final meat product have been expressed, but migration tests and risk assessment have not been well-defined in this regard. Supposed toxicity and carcinogenic claims of the use of nanoparticles on meat and food products lack sufficient proofs or verified scientific data from clinical trials, and risk assessment research limits the utilization of nanomaterials in the meat and food production industry. Therefore, an assessment of the advantages and potential risks in the use of nanotechnology to boost meat production must be well-defined. Some advantages and disadvantages in the use of nanoparticles for boosting food production as reported by Lugani et al. (2021) include the following:

Advantages of nanoparticles in food production:

1. Keep foods fresh for long duration.
2. Removal of unpleasant odor from food and provision of antimicrobial effects against potential pathogens.
3. It helps to retain volatile food condiments, dispersion, and bioavailability of food nutrients.
4. Some nanomaterials such as nanosilver give natural and potent antioxidant, antibiotic, and antibacterial properties in foodstuff.
5. Some nanocomposite materials assist to boost mechanical and rheological characteristics of foodstuffs.
6. It also helps to bind and remove possible contaminants from food.

Disadvantages of nanoparticles in food production include the following:

1. It could lead to the promotion of allergic pulmonary inflammation in humans.
2. It could cause alteration of nutrient absorption profile and metabolism in body.
3. It could result in the accumulation of various tissues and organs (such as skin, liver, lung, kidney, spleen, brain, vascular, and reproductive tissue) in the body system.
4. It could cause chromosomal damage, abnormal cellular morphology, and cell shrinkage.
5. It could also induce oxidative stress and change cell signal transduction pathways, which may lead to carcinogenesis.
6. It could trigger antigen-specific immune reactions and hypersensitivity responses.

To date, this manuscript appears to be the first scientometric study that assessed the outputs of peer-reviewed publications on nanotechnology and meat production at a global level. Although, we are aware that there might be some limitations to the present study, which may include the following:

a. Missing publications that we might not have included in the analysis of nanotechnology and meat production or its related words during the retrieval of data from WOS and Scopus.
b. Second, limitation may occur from this study since we did not include publications on nanotechnology and meat production that were in non-indexed journals and thus, would not have been available in the WOS and Scopus databases, such as those published in some Chinese or other non-English journals.
c. The present study might also be limited due to the exclusions of other document types including meeting abstracts, review articles, note papers, etc.
d. The scientometric analysis of the present paper did not reflect fully the content of the articles analyzed.

## Future Perspectives of the Utilization of Nanotechnology to Boost Meat Production

Looking at the possible future perspectives of the utilization of nanoparticles for meat production, the scientific study of the reactivity of products from nanoparticles calls for a major concern. Thorough research of the interactions that exist between the nanomaterials and the biological system of animals should be under serious check, particularly with respect to health-related issues. In addition, appropriate scientific trials should be carried out before a final decision on the approval of any use of nanoparticle. It is also very essential for further extensive studies in the field of nanotherapy and more energies should be channeled on global exploits on this technology for improved healthcare of humanity. The use of materials such as extracts and oils from plants to replace the use of chemicals/solvents from nanomaterials has a potential to lower any possible chance of harm/toxicity to animals. The use of medicinal and aromatic plants could be an effective technique for the efficient delivery of different phytomolecules and also in the synthesis of novel nanomaterials and this may provide unique prospects in the diverse application of nanotechnology in the area of healthcare. According to Kumari et al. (2019), the application of the mixture of medicinal and aromatic plants with nanotechnology could better support its application in reducing any potential health risk when considering the area of healthcare.

## Conclusions

Our scientometric analysis revealed a global increase in the use of nanotechnology techniques for meat production, with greater research output from high-income countries when compared with low- and middle-income countries and limited collaboration with developing countries. The low-research outputs in developing countries on the current subject matter mirrored similar occurrence of outputs in other research fields. Likewise, the current study revealed the need for more future studies on the use of nanotechnology to improve other meat products such as beef, mutton, chevon, etc. There is also a need for more robust studies guided by narrative reviews/meta-analysis in the future that will focus on the emerging themes and recent research directions on nanotechnology and meat production, since it is a bit challenging to recognize these emerging themes and recent research by using scientometric studies alone due to low frequency of appearance of keywords from this analysis.

## Data Availability Statement

The raw data supporting the conclusions of this article will be made available by the authors, without undue reservation.

## Author Contributions

EI: conceptualization, data curation, analysis, visualization, writing original draft, and manuscript editing. YH: logistics and supervision. Both authors contributed to the article and approved the submitted version.

## Conflict of Interest

The authors declare that the research was conducted in the absence of any commercial or financial relationships that could be construed as a potential conflict of interest.

## Publisher's Note

All claims expressed in this article are solely those of the authors and do not necessarily represent those of their affiliated organizations, or those of the publisher, the editors and the reviewers. Any product that may be evaluated in this article, or claim that may be made by its manufacturer, is not guaranteed or endorsed by the publisher.
